# Sphingosine‐1‐phosphate‐lyase deficiency affects glucose metabolism in a way that abets oncogenesis

**DOI:** 10.1002/1878-0261.13300

**Published:** 2022-08-16

**Authors:** Sumaiya Y. Afsar, Shah Alam, Carina Fernandez Gonzalez, Gerhild van Echten‐Deckert

**Affiliations:** ^1^ LIMES Institute for Membrane Biology and Lipid Biochemistry University Bonn Germany

**Keywords:** aerobic glycolysis, autophagy, cancer, HIF‐1, S1P‐lyase, sphingosine‐1‐phosphate

## Abstract

Sphingosine‐1‐phosphate (S1P), a bioactive signaling lipid, is involved in several vital processes, including cellular proliferation, survival and migration, as well as neovascularization and inflammation. Its critical role in the development and progression of cancer is well documented. The metabolism of S1P, which exerts its effect mainly via five G protein‐coupled receptors (S1PR_1–5_), is tightly regulated. S1P‐lyase (SGPL1) irreversibly cleaves S1P in the final step of sphingolipid catabolism and exhibits remarkably decreased enzymatic activity in tumor samples. In this study, we used SGPL1‐deficient (*Sgpl1* ^*−/−*^) mouse embryonic fibroblasts (MEFs) and investigated the impact of S1P on glucose metabolism. Accumulated S1P activates, via its receptors (S1PR_1–3_), hypoxia‐inducible factor 1 and stimulates the expression of proteins involved in glucose uptake and breakdown, indicating that *Sgpl1* ^*−/−*^ cells, like cancer cells, prefer to convert glucose to lactate even in the presence of oxygen. Accordingly, their rate of proliferation is significantly increased. Activation of the Akt/mTOR pathway and hence down‐regulation of autophagy indicate that these changes do not negatively affect the cellular energy status. In summary, we report on a newly identified role of the S1P/S1PR_1–3_ axis in glucose metabolism in SGPL1‐deficient MEFs.

## Graphical abstract

The human gene encoding S1P‐lyase (*SGPL1*), the enzyme that irreversibly cleaves sphingosine‐1‐phosphate (S1P) maps to a region that is prone to mutations in cancer. We show that pathological accumulation of S1P due to its deficient degradation triggers oncogenesis by stimulating glucose uptake and promoting aerobic glycolysis. As a result, HIF and Akt are activated and cell proliferation is significantly increased.

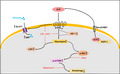

**Abbreviations**
G6P: glucose‐6‐phosphate
GAPDH: glyceraldehyde‐3‐phosphate dehydrogenase
GLUT1: glucose transporter 1
GPCR: G‐protein coupled receptor
HIF‐1: hypoxia‐inducible factor 1
IDH: isocitrate dehydrogenase
LC3: 1A/1B‐light chain 3
LDH: lactate dehydrogenase
MEFs: mouse embryonic fibroblasts
mTOR: mechanistic target of rapamycin
p62/SQSTM1: sequestosome 1
PDH: pyruvate dehydrogenase
PFK: phosphofructokinase
S1P: sphingosine‐1‐phosphate
SK: sphingosine kinase
SPGL1: S1P‐lyase

## 1 Introduction

Sphingolipids are ubiquitous components of cellular membranes [1]. In addition to their structural functions, sphingolipids also emerged as intra‐ and extracellular signaling molecules [2]. In particular, their lipid membrane anchor ceramide, [3] and its degradation product sphingosine‐1‐phosphate (S1P) [4] have multiple physiological functions. For example, S1P can bind to a family of five G‐protein coupled receptors (GPCRs) designated as S1PR_1–5_ which, depending on the coupled G proteins, can elicit several effector functions [5]. The dynamic balance between ceramide associated with apoptosis and S1P mediating cell survival, known as ‘sphingolipid rheostat’ appears straightforward, yet it is an over simplification when considering the versatile cellular functions of these two metabolites [6]. Things are additionally complicated by the fact that these two molecules are closely interconvertible. Thus, S1P is generated by means of two enzymatic reactions, deacylation of ceramide to sphingosine catalyzed by ceramidases [7] and the phosphorylation of the generated sphingoid base to S1P by sphingosine kinases (SKs). SK1 and SK2 are two kinase isoforms involved in S1P production [8]. Despite different intracellular locations, biochemical properties, and biological functions, both, SK1 and SK2 are predictive markers in inflammatory diseases and cancer [9]. This indicates, on the one hand, a key role of S1P in cell growth, survival and invasion, but also the importance of enzymes catalyzing its degradation. There are multiple enzymes involved in the catabolism of S1P. S1P phosphatases (SPPs) catalyze the removal of the phosphate group yielding sphingosine, which in turn can be either re‐phosphorylated to S1P or directed into the salvage pathway for the synthesis of ceramide [10]. The irreversible breakdown of S1P to phosphoethanolamine and hexadecenal is catalyzed by the enzyme S1P‐lyase (SGPL1) [11]. Intriguingly, the human gene encoding SGPL1 maps to a region that is prone to mutations in cancer [12, 13]. Accordingly, interference with SGPL1 expression and activity confers resistance to chemotherapy and promotes carcinogenesis [14, 15, 16]. We have recently shown that SGPL1 depletion substantially affects sphingolipid metabolism in non‐differentiated mouse embryonic fibroblasts (MEFs) in a way that might support carcinogenic potency [17]. In the present study we asked whether SGPL1 depletion also affects other metabolic pathways that, like sphingolipid metabolism, are closely connected to the development and progression of cancer [18, 19]. Aerobic glycolysis has long been associated with cell growth and cancer [20]. Since the 1950s, it has been shown that in contrast to healthy cells that convert pyruvate into lactate only in the absence of oxygen, malignant cells preferentially convert pyruvate into lactate even in the presence of oxygen [21]. Notably, a critical role in the regulation of aerobic glycolysis in carcinogenesis has been attributed to the mechanistic target of rapamycin (mTOR) [22]. The latter coordinates cell growth and metabolism with nutrient input [23]. mTOR is also a key regulator of autophagy, a fundamental cellular process that secures survival during critical circumstances including nutritional deficiency, hypoxia and other stressful conditions [24]. Thus, deregulation of mTOR is implicated in several pathologies including cancer, diabetes, neurological disorders as well as the aging process [23]. The function of aerobic glycolysis is, however, not limited to supporting cell proliferation [20]. Increased proliferation of malignant cells also implies an elevated uptake and breakdown of nutrients [25]. Thus cancer cells not only preferentially break down glucose to produce lactate even in normoxic conditions [26, 27] but also increase the rate of glucose uptake by up‐regulating the high‐affinity glucose transporter (GLUT)1 [28]. Furthermore, the role of tricarboxylic acid (TCA) cycle, as the central hub of energy and metabolite supply, emerges in cancer metabolism [29]. Hence, a growing number of studies report on the importance of an aberrant TCA cycle function in tumorigenesis. It has been shown on the one hand that cancer cells uncouple glycolysis from the TCA cycle [27, 30] and on the other hand that different enzymes of the TCA cycle are deregulated in human cancers [31].

In the present study we show that S1P accumulation as a result of SGPL1 deficiency leads to a receptor‐mediated increase of glucose uptake and its preferential breakdown to lactate. We show further that these changes of glucose metabolism are accompanied by Akt/mTOR‐mediated compromised autophagy.

## 2 Materials and methods

### 2.1 Reagents

Monoclonal antibodies against mTOR (2972S), LC3 (12741S), p62 (5114S), PDH (3205S), GAPDH (5174S), PFK (8164S), LDH (2012S), Akt (9272S), p‐Akt (193H12) and β‐actin (4967S) were purchased from Cell Signaling Technology (Danvers, MA, USA), while those against GLUT1 and p‐mTOR were from Invitrogen (Carlsbad, CA, USA; MA5‐31960 and12‐9718‐41, respectively), and those against IDH from Sigma‐Aldrich (HPA007831; St. Louis, MO, USA). Anti HIF‐1α antibody was purchased from Santa Cruz Biotechnology (Dallas, TX, USA; sc‐13 515). Secondary antibodies were HRP‐linked anti‐rabbit and anti‐mouse IgG from Cell Signaling Technology (7074S and 7076S respectively, Danvers, MA, USA). Inhibitors VPC‐23019 and rapamycin were procured from Cayman Chemical Company (Ann Arbor, MI, USA), while JTE‐013 was purchased from Sigma‐Aldrich. Akt inhibitor (Aktin‐1/2), Akt1/2 kinase inhibitor was obtained from Abcam (Cambridge, UK; ab142088). Glucose‐6‐phosphate (G6P) assay kit for enzymatic determination of G6P was purchased from Merck (Darmstadt, Germany; MAK014). FTY‐720 was from Cayman Chemical Company while MTT Assay Kit was from Abcam (ab197010).

### 2.2 Cell culture

Mouse embryonic fibroblasts [wild‐type (WT) controls and SGPL1‐deficient, *Sgpl1* ^*−/−*^, KO] were initially provided by P. P. Van Veldhoven (KU Leuven, Leuven, Belgium) and cultured as described previously [32]. Briefly, cells were maintained in Dulbecco's Modified Eagle Medium (Gibco™, Paisley, UK; 31966) containing 10% fetal bovine serum (PAN Biotech, Aidenbach, Germany; P40‐47100) supplemented with 100 units·mL^−1^ penicillin and 100 mg·mL^−1^ streptomycin (Gibco™ [Life Technologies, Darmstadt, Germany]; 15140122). Cells were grown in 25 cm^2^ (T25) flasks and maintained in a humidified incubator at 37 °C with 5% CO_2_. The cells were passaged every 2–3 days prior to confluence and harvested by trypsinization using 0.05% Trypsin–EDTA (Sigma‐Aldrich, Taufkirchen, Germany; 59418C). All experiments were performed at 70–80% confluency of the cell layer.

### 2.3 Western immunoblotting

Cell samples were homogenized in RIPA buffer (Thermo Fisher Scientific, Rockford, IL, USA; 89900). Samples were kept on ice for 1 h with intermittent pipetting followed by centrifugation at 20 000 *g* at 4 °C for 45 min. Nanodrop (Thermo Fisher Scientific; ND‐2000) was used to determine the protein concentration in the supernatants. Lysates were mixed with Laemmli buffer in a 1 : 4 ratio (Bio‐Rad Laboratories, Munich, Germany; 1610747), and samples were heated for 5 min at 95 °C before loading on SDS/PAGE gel. Proteins were separated by SDS/PAGE in running buffer (25 mm Tris, pH 8.3, 192 mm glycine, 0.1% SDS) at 50 V for 15 min, then 1 h at 150 V. Transfer onto nitrocellulose membranes (Immobilon‐P, IPVH00010; Merck) was performed at 4 °C and 400 mA for 2 h in transfer buffer (50 mm Tris, pH 9.2, 40 mm glycine, 20% methanol). Membranes were blocked with Blocker BSA (Thermo Scientific; 37520) in TBS‐Tween 20 (20 mm Tris, pH 7.5, 150 mm NaCl, 0.1% Tween 20, P9416; Sigma‐Aldrich) for 1 h, washed three times (10 min each) and incubated at 4 °C overnight with the primary antibody. Membranes were then washed three times (10 min each) and incubated for 1 h at room temperature with an HRP‐conjugated secondary antibody. Western BLoTChemiluminescence HRP Substrate (TAKARA Bio, Saint‐Germain‐en‐Laye, France; T7101B) was used for detection with the VersaDoc 5000 imaging system (Bio‐Rad, Hercules, CA, USA). β‐actin was used as the loading control. Quantification and statistical analysis were performed using imagej (ImageJ 1.51j8, NIH, Bethesda, MD, USA) and graphpad prism program (Graphpad Software, San Diego, CA, USA).

### 2.4 Glucose‐6‐phosphate determination

Glucose‐6‐phosphate was determined by colorimetric detection at 450 nm using the G6P Assay kit (Merck; MAK014). The total protein concentration of each sample was used as a reference. The deproteination step was performed by adding an equal volume of ice‐cold 0.5 m HClO_4_ and incubating on ice for 5 min [33]. Thereafter to remove protein, the mixture was centrifuged at 10 000 ***g*** for 5 min, and the supernatant was collected. Two hundred microlitre of the supernatant was neutralized with 10 μL of 2.5 m K_2_CO_3_ at 4 °C. Samples were further degassed and briefly centrifuged for 5 min. A clear supernatant was collected and used for the G6P assay following the instructions provided by the manufacturer.

### 2.5 RNA isolation and quantitative real‐time PCR

Total RNA was extracted using the EXTRAzol kit (Blirt, Gdańsk, Poland; EM30–200) according to the manufacturer's instructions. Reverse transcription was performed using the ProtoScript® II First Strand cDNA Synthesis kit (New England Biolabs, Ipswich, MA, USA; E6560L). The resulting total cDNA was then applied in real‐time PCR (CFX96‐real‐time PCR; Bio‐Rad Laboratories) to measure mRNA using β‐actin as the housekeeping gene. The primers for real‐time PCR were designed using the online tool from NCBI blast and procured from Invitrogen. They are listed as follows; β‐actin: (forward) 5′‐CTTTGCAGCTCCTTCGTTGC‐3′, (reverse) 3′‐CCTTCTGACCCATTCCCACC‐5′; S1PR1: (forward) 5′‐CTACACAACGGGAGCAACAG‐3′, (reverse) 3′‐CCCCAGGATGAGGGAGAGAT‐5′; S1PR2: (forward) 5′‐CAGGATCTACTCCTTGGTCAGG‐3′, (reverse) 3′‐GAGATGTTCTTGCGGAAGGT‐5′; S1PR3: (forward) 5′‐CCCAACTCCGGGACATAGA‐3′, (reverse) 3′‐ACAGCCAGTGGTTGGTTTTG‐5′; S1PR4: (forward) 5′‐TTCCATATGATGGACACTCC‐3′, (reverse) 3′‐TGGACAAATGAACGCAGGT‐5′; S1PR5: (forward) 5′‐GCTTTCTGTGTACAGTTGACAAATACT‐3′, (reverse) 3′‐CCAACTGTTCCAACTGTATGCT‐5′. The reactions were performed at 95 °C for 30 s, 95 °C for 5 s, 60 °C for 1 min.

### 2.6 Cell proliferation

The proliferative potential of MEFs (WT and KO cells) was monitored using the MTT (3‐(4,5‐Dimethylthiazol‐2‐yl)‐2,5‐Diphenyltetrazolium Bromide) assay from Abcam (ab197010). About 5 × 10^3^ cells per well were seeded in 96 well plates in DMEM containing 10% fetal bovine serum and cultured for 24 h. Then the medium was changed, and 50 μL of MTT reagent was added in 50 μL of serum‐free DMEM as indicated by the provider, and the plate was kept in the incubator for 3 h to form the formazan crystals. The medium with MTT reagent was then discarded, and the experiment was terminated by adding 150 μL of MTT solvent in each of the wells. The 96‐well plate was kept on the shaker for 10–15 min so that the crystals were completely dissolved. Absorbance was recorded at 590 nm using FLUOstar Omega (BMG LABTECH, Ortenberg, Germany). Data obtained are expressed relative to the WT controls.

### 2.7 Statistical analysis

For statistical analysis, graphpad prism 9 software was used. Each result is expressed as means ± SEM based on at least three independent experiments if not otherwise stated. The significance of differences between the experimental groups and controls was assessed by either unpaired Student *t*‐test or one‐way analysis of variance (ANOVA) with Tukey's *post hoc* correction, as appropriate. *P* < 0.05 was considered statistically significant (*P < 0.05; **P < 0.005; ***P < 0.0005; ****P < 0.00005; compared with the respective control group).

## 3 Results

### 3.1 Glucose uptake and metabolism is altered in SGPL1‐deficient MEFs

**Fig. 1:**
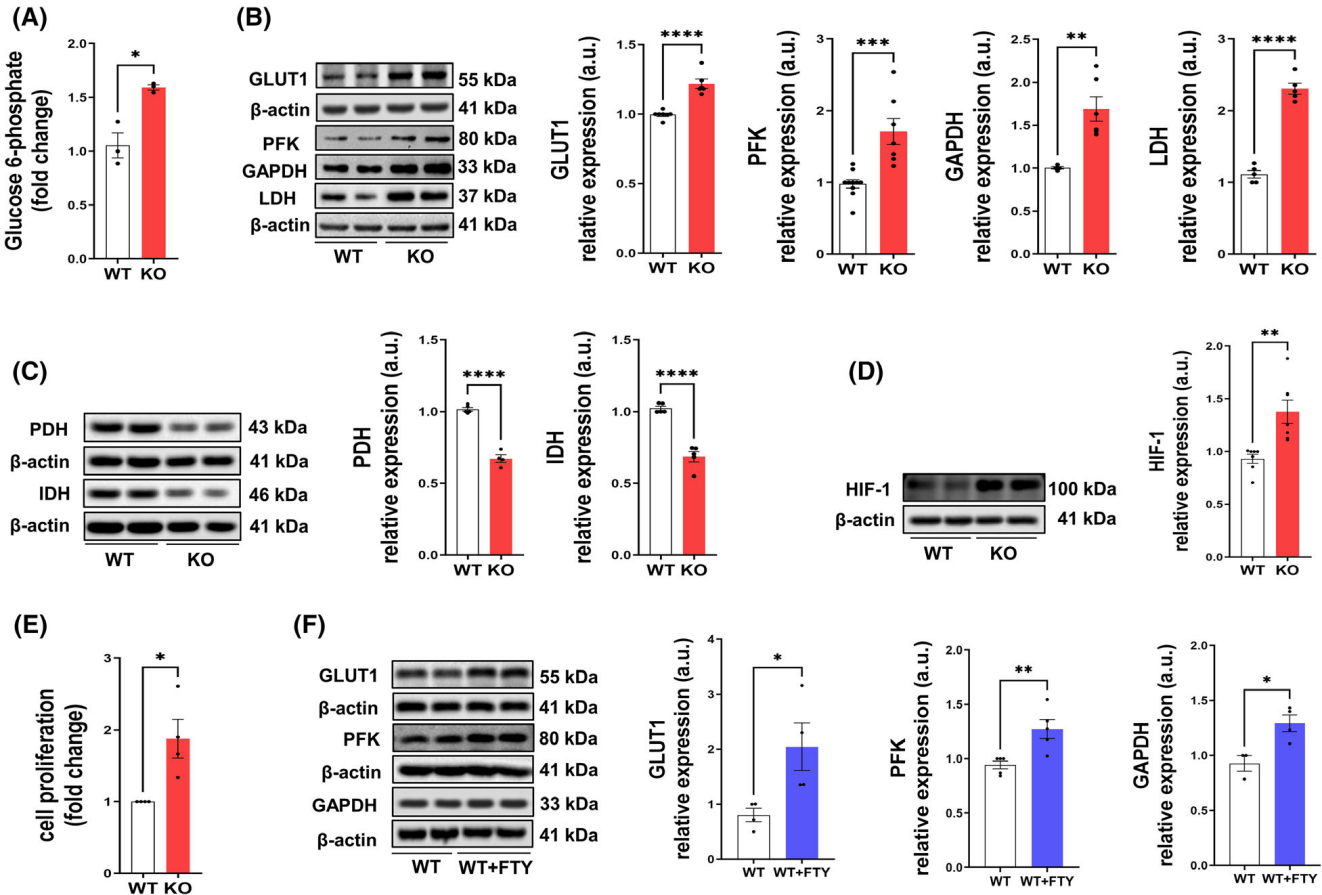
Remodeled glucose metabolism in SGPL1‐deficient MEFs. (A) Colorimetric determination of G6P shows increased levels in KO (red bars) relative to WT (white bars) MEFs. (B–D) Protein quantification of GLUT1, PFK, GAPDH, LDH, PDH, and IDH and HIF‐1 in WT controls (white bars) and SGPL1‐deficent (KO) MEFs (red bars). (E) Rates of proliferation of KO (red bars) are increased relative to WT (white bars) MEFs. (F) Protein quantification of GLUT1, PFK, and GAPDH in WT MEFs cultured in the absence or presence of 10 nm FTY720 (FTY) for 24 h. Shown is one representative western blot for each protein. β‐Actin was used as loading control. Bars represent means ± SEM (*n* ≥ 3, ****P < 0.00005, ***P < 0.0005, **P < 0.005, *P < 0.05; unpaired student *t*‐test). a.u., arbitrary units; HIF‐1, hypoxia‐inducible factor 1.

It has been shown previously that S1P levels increase significantly in MEFs lacking SGPL1 activity [17, 32, 34]. Here, we first assessed the effect of SGPL1 depletion (Fig. S1) on glucose uptake and metabolism. The common cellular form of glucose is G6P, as the majority of glucose entering a cell is phosphorylated at the hydroxyl group on carbon 6. As shown in Fig. 1A, the levels of G6P were significantly higher (by about 30%) in SGPL1 knock‐out (KO) cells than in wild‐type (WT) controls. The reason for an increased level of G6P in *Sgpl1* ^*−/−*^ cells could be explained either by a higher uptake of the hexose or by its decreased metabolization. We, therefore, next investigated the highly sensitive glucose transporter GLUT1. We found that GLUT1 accumulated significantly in*Sgpl1* ^*−/−*^ MEFs compared with WT controls (Fig. 1B), suggesting an increased uptake of glucose into KO cells. Then we examined the expression of two glycolytic enzymes: (a) phosphofructokinase (PFK), the rate‐limiting enzyme of glycolysis and (b) glyceraldehyde‐3‐phosphate dehydrogenase (GAPDH), which due to its constant expression in most cell types is often used as a housekeeping reference [35]. As illustrated in Fig. 1C the protein levels of both enzymes are elevated by about 40% in SGPL1‐deficient MEFs, indicating an up‐regulated glycolytic degradation of glucose in these cells. To explore the fate of pyruvate, the end product of glycolysis, we analyzed both lactate dehydrogenase (LDH), which converts pyruvate into lactate usually in the absence of oxygen, and pyruvate dehydrogenase (PDH), which catalyzes its oxidative decarboxylation into acetyl‐coenzyme A feeding the TCA cycle. Surprisingly, the expression of LDH was substantially (twofold) increased (Fig. 1C), while that of PDH was significantly decreased by nearly 40% (Fig. 1D) in *Sgpl1* ^*−/−*^ MEFs. To obtain additional information concerning TCA cycle turnover, we examined the expression of isocitrate dehydrogenase (IDH), one of the TCA cycle enzymes that is often found to be mutated in several cancers [36]. Like PDH, IDH was also decreased by almost 40% (Fig. 1D). Together, these results suggest that SGPL1‐deficient cells bypass the TCA cycle and primarily degrade glucose via aerobic glycolysis instead. One cause of aerobic glycolysis in cancer is the activation of hypoxia‐inducible factor 1 (HIF‐1) [37]. As shown in Fig. 1D the expression of this transcription factor is significantly elevated by about 30% in SGPL1‐deficient MEFs (Fig. 1D). We then monitored the proliferation rate of *Sgpl1* ^*−/−*^ MEFs and found that it is almost doubled compared with WT MEFs (Fig. 1E). Finally, in support of our assumption that the effects on glucose metabolism are S1P‐dependent, we treated WT MEFs for 24 h with the S1P receptor agonist FTY720 (10 nm) and recapitulated the measurements of GLUT1 and the glycolytic enzymes. The results obtained resemble those of *Sgpl1* ^*−/−*^ MEFs indicating that the effects seen in SGPL1‐deficient MEFs are indeed caused by S1P (Fig. 1F).

### 3.2 S1P stimulates glucose uptake and aerobic glycolysis via S1PR_1_ _–3_

**Fig. 2:**
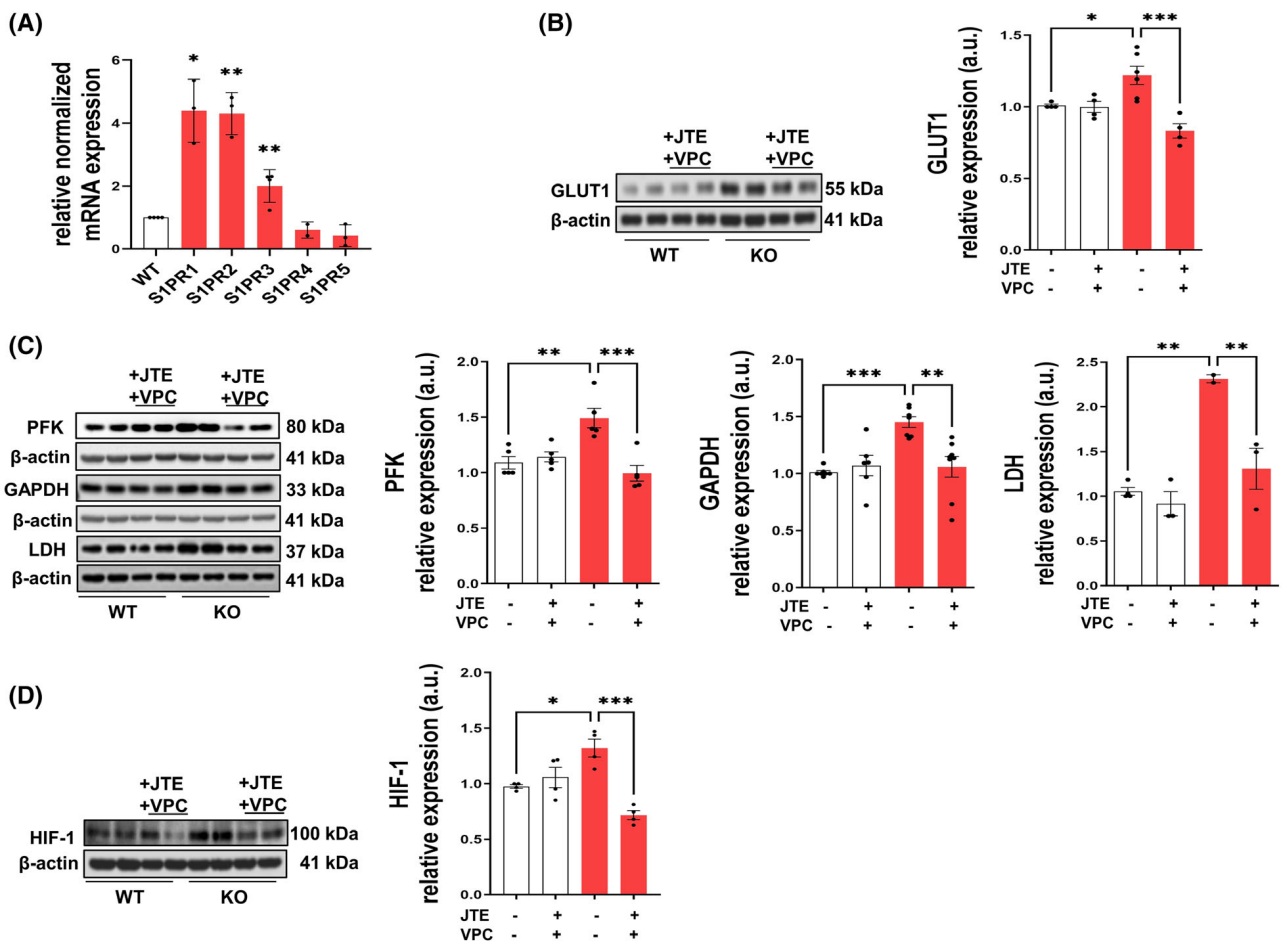
S1PR_1–3_ mediate the effect of S1P on remodeled glucose metabolism in SGPL1‐deficient MEFs. (A) Transcript amounts of S1PRs were evaluated by quantitative real time PCR in SGPL1‐deficient (KO) MEFs (red bars) relative to WT controls (white bar) as indicated. Bars represent means ± SEM (*n* ≥ 3, **P < 0.005, *P < 0.05; Student *t*‐test). (B, C) Protein quantification of (B) GLUT1 and of (C) PFK, GAPDH, and LDH and of (D) HIF‐1 in WT controls (white bars) and SGPL1‐deficent (KO) MEFs (red bars) in the presence or absence of VPC‐2309 and JTE‐013 as indicated. Shown is one representative western blot for each protein. β‐Actin was used as loading control. Bars represent means ± SEM (*n* ≥ 3, for HIF‐1 *n* = 2, ***P < 0.0005, **P < 0.005, *P < 0.05, one‐way ANOVA with Tukey's *post hoc* correction). a.u., arbitrary units; HIF‐1, hypoxia‐inducible factor 1.

In view of the fact that S1P is known to carry out most of its functions via a family of five GPCRs, known as S1PR_1–5_, it was important to investigate their potential role in the modified glucose metabolism found in SGPL1‐deficient MEFs. We therefore began by examining the expression of S1PR_1–5_ on a transcriptional level in WT and KO MEFs by quantitative RT‐PCR. Among the five receptors, mRNA expression of S1PR_1_ and S1PR_2_ was found to be increased up to fourfold and that of S1PR_3_ by about twofold in KO MEFs, while no significant changes were detectable for S1PR_4_ and S1PR_5_ (Fig. 2A). To find out whether the described effects on glucose uptake and degradation via aerobic glycolysis are mediated by these receptors, we cultured MEFs for 24 h in the presence of VPC‐23019 (10 μm), a competitive antagonist S1PR_1_ and S1PR_3,_ and of JTE‐013 (10 μm), a specific S1PR_2_ antagonist. Then we analyzed the expression level of the proteins involved in glucose uptake and metabolism. First of all, we measured the expression of GLUT1 in the S1PR_123_‐inhibited cells and found that the elevated expression of GLUT1 was reversed to control levels in SGPL1‐deficient cells, while S1PR antagonists did not affect the expression level of GLUT1 in WT controls (Fig. 2B). Similar results were obtained for PFK, GAPDH, LDH as well as of the transcription factor HIF‐1. Specific inhibition of S1PR_1–3_ reversed the effect of SGPL1 deficiency by bringing the expression level of the three enzymes and of HIF‐1 back to control values or even below (Fig. 2C,D). However, S1PR antagonists did not reverse the diminished expression of PDH and IDH detected in SGPL1‐deficient MEFs (result not shown). Note that separate inhibition of each S1PR had no rescue effect indicating that at least concerning glucose metabolism the three S1PRs replace one another. Taken together, these data strongly point towards an S1P‐induced and S1PR_1–3_‐mediated molecular mechanism responsible for the increased glucose uptake and its degradation via aerobic glycolysis in SGPL1‐deficient MEFs.

### 3.3 mTOR‐dependent autophagy is decreased in SGPL1‐deficient MEFs

**Fig. 3:**
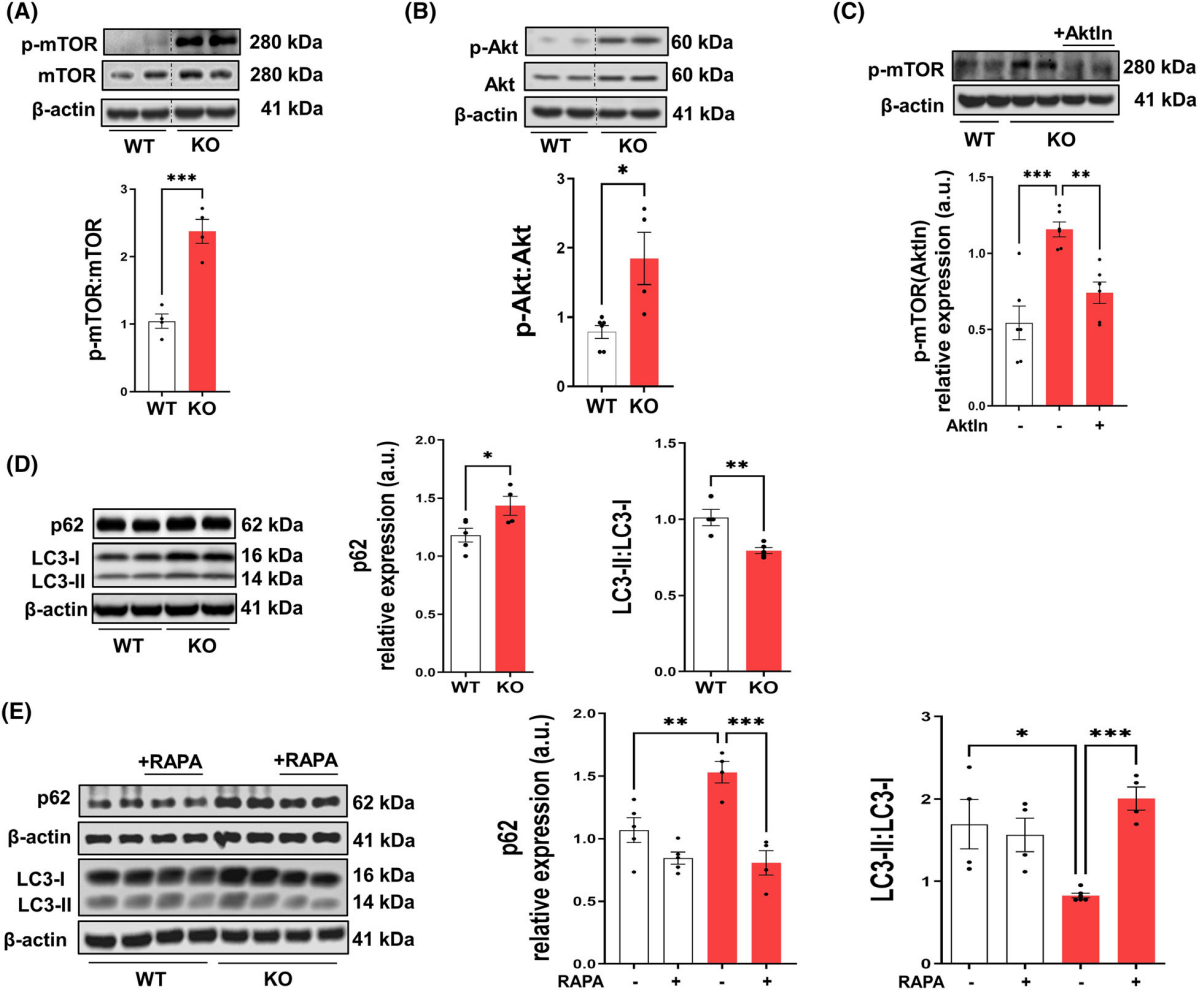
mTOR‐dependent autophagy is decreased in SGPL1‐deficient MEFs. Quantification of (A) p‐mTOR and of mTOR, (B) the ratio p‐Akt : Akt (D) p62, and the ratio LC3‐II : LC3‐I in WT controls (white bars) and SGPL1‐deficent (KO) MEFs (red bars). Bars represent means ± SEM (*n* ≥ 3, ***P < 0.0005, **P < 0.005, *P < 0.05 unpaired Student *t*‐test). (C) Quantification of p‐mTOR in the absence (−) and presence (+) of AktIn (Akt inhibitor) in controls (WT, white bars) and in SGPL1‐deficient (KO) MEFs (red bars). Bars represent means ± SEM (*n* ≥ 3, ***P < 0.0005, **P < 0.005 one‐way ANOVA with Tukey's *post hoc* correction). (E) Quantification of p62, and of the ratio LC3‐II : LC3‐I in the absence (−) and presence of rapamycin (RAPA) in controls (WT, white bars) and in SGPL1‐deficient (KO) MEFs (red bars). Bars represent means ± SEM (*n* ≥ 3, ***P < 0.0005, **P < 0.005, *P < 0.05, one‐way ANOVA with Tukey's *post hoc* correction). For all one representative western blot is shown. β‐Actin was used as the loading control. a.u., arbitrary units; p‐mTOR, phospho mTOR; Akt, protein kinase B; p‐Akt, phospho‐Akt; p62/SQSTM1, sequestosome 1.

As mTOR plays a critical role in the regulation of aerobic glycolysis [22], we investigated the expression of mTOR as well as of its phosphorylated activated form p‐mTOR in WT and SGPL1‐deficient MEFs. As shown in Fig. 3A we found that the ratio p‐mTOR : mTOR is more than twofold elevated in SGPL1‐deficient MEFs (Fig. 3A). In general, high levels of mTOR are indicative of anabolic processes which are promoted by the PI3K/Akt pathway [38]. We therefore examined activation of Akt in SGPL1‐deficient MEFs. As depicted in Fig. 3B the level of activated Akt was by nearly 2 times higher in *Sgpl1* ^*−/−*^ cells than in controls. Moreover, inhibition of Akt with the specific inhibitor AktIn (5 μm, 24 h) reversed mTOR phosphoarylation bringing it nearly back to control levels (Fig. 3C), supporting the idea that S1P stimulates anabolic processes. Note that addition of AktIn did not abolish the increased expression neither of GLUT1 nor of PFK and GAPDH (not shown), excluding an activation of glucose degradation by Akt/mTOR signaling.

Given the multifaceted role of mTOR as master regulator of autophagy [39], we next analyzed two autophagy marker proteins: (a) p62/sequestosome, a specific autophagy substrate, which identifies and confiscates autophagy cargo, and (b) 1A/1B‐light chain 3 (LC3). The latter exhibits two forms, LC3‐I, which after lipidation to LC3‐II is conjugated to the growing autophagosomal vesicle thus, supporting its elongation and maturation. As shown in Fig. 3D, immunoblots reveal a slight but significant increase of p62 expression and a drop of the ratio of LC3‐II : LC3‐I in MEFs lacking SGPL1 (Fig. 3D). These results point to a significant decline of autophagy in SGPL1‐deficient MEFs. Treatment of cells with rapamycin (1 μm) for 24 h restored autophagy in SGPL1‐deficient MEFs as shown by the reestablished amount of p62 and the normalized ratio of LC3‐II : LC3‐I (Fig. 3E). This result confirmed that impaired autophagy in SGPL1‐deficient MEFs is mediated by the increased expression of mTOR. Of note, addition of rapamycin did not abolish the increased expression of PFK and GAPDH, (not shown) confirming once more that the increased levels of p‐mTOR as well as the activation of Akt are not responsible for the elevated levels of glycolytic enzymes.

## 4 Discussion

**Fig. 4:**
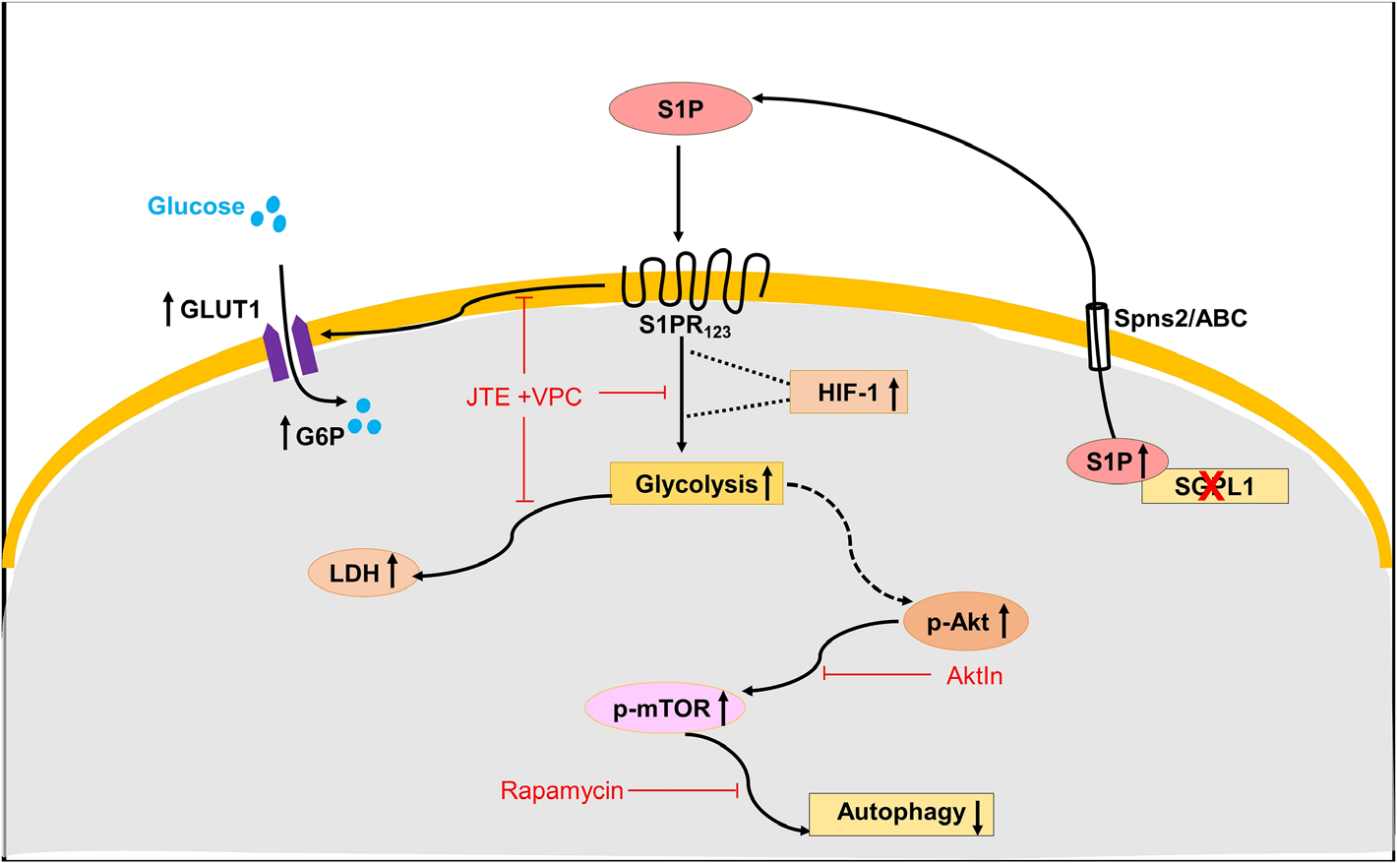
Scheme summarizing the effects of SGPL1 ablation in MEFs. In the absence of SGPL1, accumulated S1P is secreted by the cells [55]. By binding to S1PR_1–3_, it elicits signaling cascades that promote increased expression of proteins involved in glucose uptake and breakdown via aerobic glycolysis. This and the high energy state of the cells promote activation of Akt/mTOR pathway leading to decreased autophagy. Shown are the sites affected by the S1PR antagonists VPC and JTE as well as the Akt inhibitor AktIn and the mTOR inhibitor rapamycin. See text for further explanations. ABC, ATP‐binding cassette transporters; HIF‐1, hypoxia‐inducible factor 1; p‐Akt, phospho‐Akt; p‐mTOR, phospho‐mTOR; Spns2, spinster 2.

We have reported recently that depletion of SGPL1 in MEFs affects sphingolipid metabolism in a way that is characteristic of cancer cells [17]. Moreover, the lack of SGPL1 has been associated with increased cell proliferation *in vitro* and oncogenesis *in vivo* [15]. Hence, we postulated that accumulation of S1P in MEFs due to SGPL1 deficiency might also affect glucose metabolism in a way that promotes carcinogenesis. Consistently, we found an increase of glucose uptake and a shift to aerobic glycolysis in SGPL1‐deficient cells, two candidates of tumor progression [26, 27]. Accordingly, the expression of the high‐affinity GLUT1 and of LDH is substantially increased in SGPL1‐deficient MEFs. Furthermore, the significant increase of PFK, the rate‐limiting enzyme of glycolysis as well as of GAPDH, one of the most commonly used housekeeping proteins [35], indicates an elevated glycolytic breakdown of glucose in SGPL1‐deficient cells. Our results indicate that HIF‐1, generally known to orchestrate cellular adaptation to low oxygen, thus promoting malignancy of cancer cells [40], also mediates aerobic glycolysis in SGPL1‐deficient MEFs (Fig. 4). But HIF‐1 is also a major component of the embryo development and plays a central role in normal cellular functions and in tissue metabolism [41]. The S1P/S1PR_1–3_‐promoted expression of HIF‐1 in SGPL1‐deficient MEFs is oxygen‐independent and could resemble HIF‐1 activation by insulin and insulin‐like growth factors (IGFs) [42]. It is well documented that growth and survival factors acting via receptor tyrosine kinases (RTKs) as well as hormones signaling via GPCRs promote the expression of genes encoding proteins that stimulate metabolic pathways in support of cell proliferation [25, 43]. Our results regarding glucose uptake and breakdown via aerobic glycolysis support the notion that S1P promotes via its GPCRs the expression of genes including *Glut1* and of *Ldh* known to be highly expressed in proliferating cancer/malignant cells [26, 27]. On the other hand, the expression of PDH, the enzyme complex catalyzing the oxidation of pyruvate to acetyl‐CoA to feed the TCA cycle as well as the expression of IDH, catalyzing oxidative decarboxylation of isocitrate to yield α‐ketoglutarate is significantly reduced in *Sgpl1* ^*−/−*^ cells. This result suggests that SGPL1‐deficient cells bypass the TCA cycle, consistent with an early yet refuted dogma regarding glucose metabolism in cancer cells [44]. Intriguingly, the expression of these two enzymes could not be rescued by treating the cells for 24 h with inhibitors of S1PR_1–3_. A possible explanation could be the fact that reprogramming of TCA cycle enzymes localized to the mitochondrial matrix may demand a longer time period than that of glycolytic enzymes in the cytosol.

We have to point out that the high level of glucose uptake and degradation via aerobic glycolysis at the expense of the TCA cycle is not necessarily equal to increased proliferation and difficult to explain from an energetic point of view. To resolve this apparent paradox, one has to envisage all metabolic requirements for proliferation that include not only ATP but also nucleotides for nucleic acids, amino acids for proteins and lipids for membranes [45]. The biosynthetic pathways that generate all these metabolites either branch out from glycolysis or use glucose as a primary source as for example the pentose phosphate path (PPP), generating ribose for nucleotides and NADPH, the reduction equivalents needed for all biosynthetic pathways and for the generation of glutathione (GSH) to protect cells against free radicals. Accordingly, proliferating fibroblasts were shown to rely on PPP with their TCA cycle being interrupted between citrate and α‐ketoglutarate [46]. However, like many cancer cells they use glutamine as an anaplerotic substrate to produce α‐ketoglutarate [44]. For more clarity regarding the energy status of SGPL1‐deficient MEFs, we investigated activation of the pathway involving Akt and mTOR. The serine/threonine protein kinase mTOR functions as an ATP and amino acid sensor hence adjusting nutrient availability and cell growth and survival [47]. It is activated by mitogen‐responsive pathways that signal energy and nutrient availability. Accordingly, activation of the PI3K/Akt pathway promoting cell proliferation and survival is considered the prototypic mechanism of mTOR regulation [48]. Therefore, activation of Akt by phosphorylation and an increased expression of mTOR in SGPL1‐deficient MEFs indicate that the metabolic changes that occurred in the absence of SGPL1 are favourable for the energy load of the cells. Furthermore, mTOR is considered a master regulator of autophagy as its inhibition with rapamycin was shown to initiate autophagy. Meanwhile it is well documented that the role of mTOR as a regulator of autophagy is rather complex and affects not only the initiation but also subsequent steps of the autophagy process [39]. In any case, the increased activation of mTOR by phosphorylation in SGPL1‐deficient MEFs argues in favour of a decline of autophagy in these cells. Consequently, our results convincingly illustrate a decrease of autophagy in *Sgpl1* ^*−/−*^ cells, which could be restored to control levels by rapamycin treatment (Fig. 4). However, in an earlier study no changes of autophagy were monitored in SGPL1‐deficient MEFs compared with their WT counterparts [15]. At present, we have no explanation for this discrepancy. But we want to point out that in the same study the authors found an up‐regulation of the antiapoptotic proteins Bcl‐2 and Bcl‐xL that protect SGPL1‐deficient MEFs against apoptosis induced by chemotherapeutic agents [15]. Intriguingly, Bcl‐2 not only functions as an antiapoptotic but also as an antiautophagy protein due to its inhibitory interaction with Beclin1, an evolutionarily conserved autophagy protein [49]. Interestingly, the antiapoptotic effect of Bcl‐2 could be prevented by rapamycin pointing towards the involvement of mTOR and of its upstream activator Akt [50]. These findings additionally support the oncogenic potential of cells lacking SGPL1. Accordingly, we monitored substantially increased proliferation rates of SGPL1‐defcient MEFs compared with WT controls. Similar, yet less distinct differences of the proliferation rates of *Sgpl1* ^*−/−*^ versus WT MEFs have been reported earlier [15].

## 5 Conclusion

The results of the present study not only confirm the tight connection of energy metabolism and autophagy [51], and the link between sphingolipid metabolism and autophagy [52, 53] but in addition comprehensively articulate the impact of sphingolipid metabolism on glucose uptake and breakdown and thereby on energy metabolism.

Last but not least, we want to mention the potential relevance of our findings for the novel childhood syndrome SPLIS caused by an insufficiency of SGPL1 [54]. The results of the present study showing the crucial role of the SGPL1/S1P/S1PR axes for essential cellular processes like energy metabolism and autophagy might also explain the extensive range of anomalies reported for SPLIS, including hydrops fetalis, immunodeficiency, acanthosis, renal and adrenal insufficiencies and neurological failures.

## Conflict of interest

The authors declare no conflict of interest.

## Author contributions

GE‐D and SA designed and supervised the experiments. SYA and CFG performed the experiments. SYA analyzed the data with assistance from SA. GE‐D wrote the manuscript. All authors read and approved the final manuscript.

### Peer review

The peer review history for this article is available at https://publons.com/publon/10.1002/1878‐0261.13300.

## Supporting information


**Fig. S1.** The expression of S1P‐lyase (SGPL1) in WT and *Sgpl1* ‐/‐ MEFs. Shown is a representative Western immunoblot. Bars represent means ± SEM, (n ≥ 3, ****p<0.00005; unpaired student t‐test).

## Data Availability

The original data generated or analyzed during this study, including the supporting information are included in the article.
