# Supplementary figures and images for: Sphingosine‐1‐phosphate‐lyase deficiency affects glucose metabolism in a way that abets oncogenesis
Source: Mol Oncol. 2022 Aug 16;16(20):3642–53. doi: 10.1002/1878-0261.13300 (PMC9580888; doi:10.1002/1878-0261.13300)

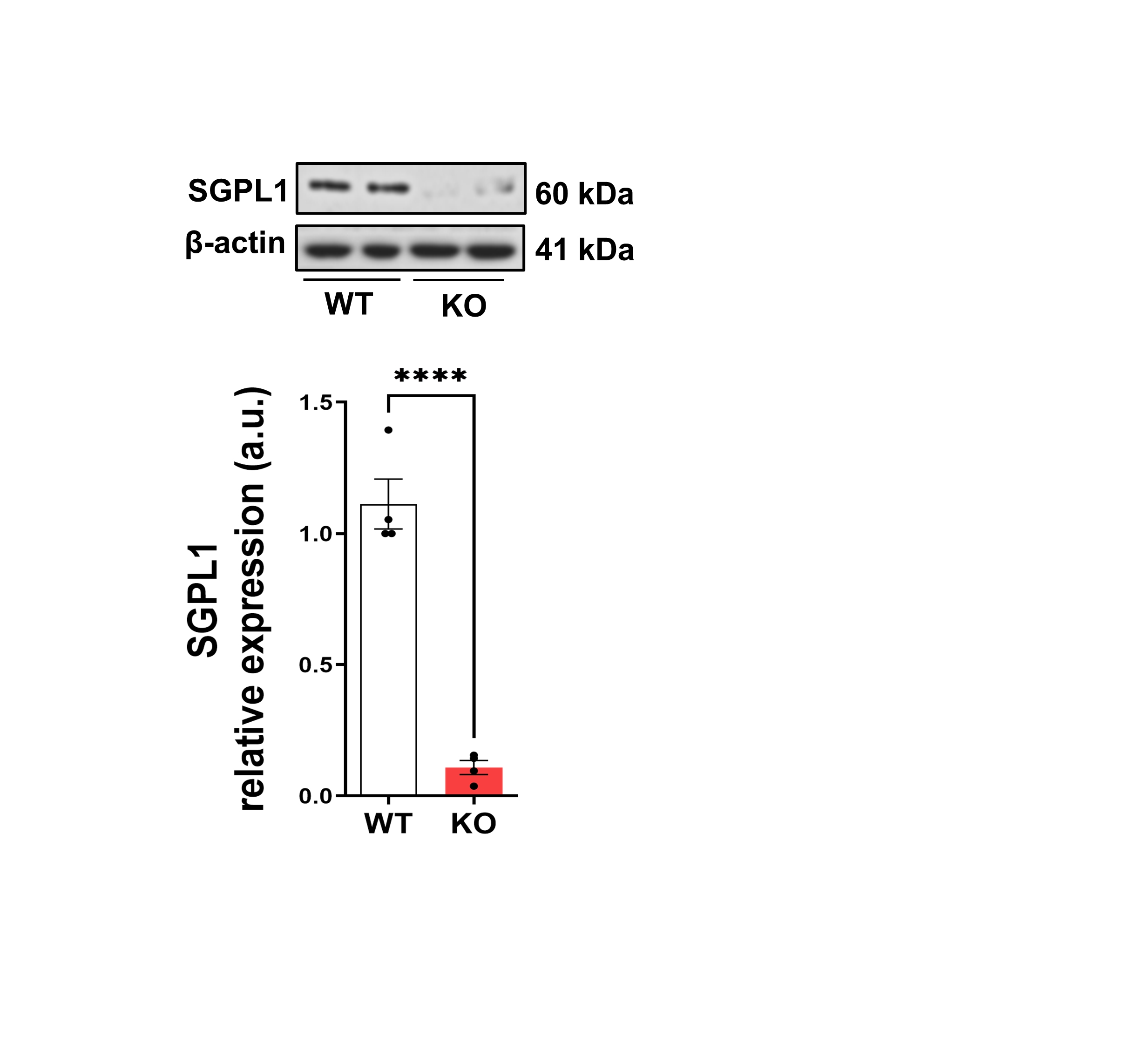

Supplement: Supplementary file 1 — Fig. S1. The expression of S1P‐lyase (SGPL1) in WT and Sgpl1 ‐/‐ MEFs. Shown is a representative Western immunoblot. Bars represent means ± SEM, (n ≥ 3, ****p<0.00005; unpaired student t‐test). [file MOL2-16-3642-s001.jpg]
